# Gestational trophoblastic disease: understanding the molecular mechanisms of placental tumours

**DOI:** 10.1242/dmm.052010

**Published:** 2025-01-28

**Authors:** Alina Nicheperovich, Benjamin Schuster-Böckler, Máire Ní Leathlobhair

**Affiliations:** ^1^ Ludwig Institute for Cancer Research, Nuffield Department of Medicine, University of Oxford, Oxford OX3 7DQ, UK; ^2^ Department of Microbiology, Trinity College, Dublin D02 VF25, Ireland

**Keywords:** Gestational trophoblastic disease, Gestational trophoblastic neoplasia, Molar pregnancy, Choriocarcinoma, Placental tumour

## Abstract

Gestational trophoblastic disease (GTD) describes a group of rare benign and cancerous lesions originating from the trophoblast cells of the placenta. These neoplasms are unconventional entities, being one of the few instances in which cancer develops from the cells of another organism, the foetus. Although this condition was first described over 100 years ago, the specific genetic and non-genetic drivers of this disease remain unknown to this day. However, recent findings have provided valuable insights into the potential mechanisms underlying this rare condition. Unlike previous reviews focused primarily on the clinical and diagnostic aspects of disease development, this Review consolidates the latest research concerning the role of genetics, epigenetics and microRNAs in the initiation and progression of GTD. By examining GTD from a molecular perspective, this Review provides a unique framework for understanding the pathogenesis and progression of this rare disease.

## Teaser

**Summary:** Gestational trophoblastic disease (GTD) encompasses rare benign and malignant placental lesions. This Review explores the potential genetic, epigenetic and microRNA drivers of GTD, highlighting insights into cancer biology, diagnostics and therapeutic opportunities.

## Introduction

**Fig. 1.**
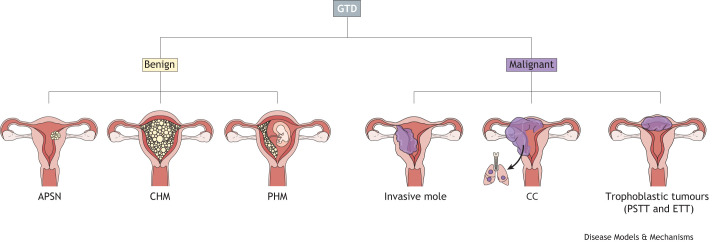
**Gestational trophoblastic disease (GTD) classification.** GTD encompasses a spectrum of benign and malignant lesions in the placenta. Benign lesions include atypical placental site nodule (APSN) and molar pregnancies [partial hydatidiform moles (PHMs) and complete hydatidiform moles (CHMs)]. Malignant gestational lesions, also known as gestational trophoblastic neoplasia (GTN), include invasive moles, choriocarcinoma (CC), placental site trophoblastic tumour (PSTT) and epithelioid trophoblastic tumour (ETT). Whereas all invasive moles follow a molar pregnancy, other cancers can be of molar or non-molar origin. The arrow indicates metastasis.

**Fig. 2.**
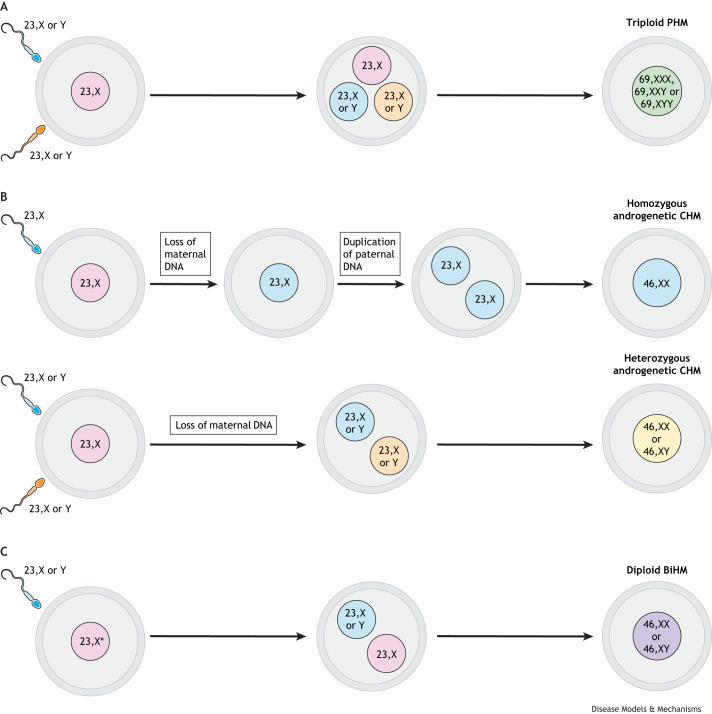
**Genetic origins of different types of hydatidiform mole**. (A) Almost all triploid PHMs form as a result of fertilisation by two sperms (dispermy). (B) Top: the majority of androgenetic CHMs are homozygous. They arise as a consequence of monospermic fertilisation where maternal chromosomes are lost either before or after conception, followed by duplication of paternal DNA. Bottom: in some cases, heterozygous CHMs form because of dispermy followed by loss of maternal DNA. (C) Most biparental hydatidiform moles (BiHMs) have a normal karyotype but exhibit a molar phenotype owing to imprinting defects in the maternal genome. Fertilisation occurs as normal. *Maternal genome is abnormally imprinted, e.g. owing to biallelic mutations in *KHDC3L* or *NLRP7*.

The human placenta, sharing the same genetic composition as the foetus, develops from the trophectoderm of the early embryo. This layer gives rise to trophoblast cells, the epithelial-like cells that form the placenta and are crucial for supporting foetal growth and development throughout pregnancy (Box 1). Gestational trophoblastic disease (GTD) arises from the uncontrolled proliferation of these trophoblast cells, which can lead to the formation of molar tissue, an abnormal mass of cells that fills the uterus, or cancer. GTD is thus broadly classified into two groups: benign and malignant (Fig. 1). These groups are then further subdivided; benign forms include the hydatidiform mole and the more recently discovered atypical placental site nodule (APSN), a benign lesion that develops from residual placental tissue in the uterus following a delivery or termination of pregnancy. Hydatidiform moles can be classified as complete (CHM) or partial (PHM), primarily based on pathological features (Fig. 1), although genotyping can be used (Fig. 2). Meanwhile, malignant lesions of the placenta are collectively called gestational trophoblastic neoplasia (GTN), which encompasses invasive moles, choriocarcinoma (CC), placental site trophoblastic tumour (PSTT) and epithelioid trophoblastic tumour (ETT) (Fig. 1) (Ngan et al., 2021). Although molar pregnancies can be treated by the removal of abnormal tissue (Sebire and Seckl, 2008), cancerous forms frequently require chemotherapy and/or hysterectomy and, in exceptionally rare cases, can metastasise to distal sites such as the brain, liver and lungs, leading to maternal death (Lurain, 2010).

The first reference to GTD dates to around 400 B.C., when Hippocrates described it as “dropsy of the uterus” (Ober and Fass, 1961). By 1895, the German pathologist F. J. Marchand had identified CC as a tumour arising from trophoblast cells, thus linking the disease to pregnancy (Marchand, 1895). Since then, there have been great advances in the clinical management of GTD, making its cancerous forms the most curable solid tumours (Seckl et al., 2010). However, much less is understood about the pathology of GTD from a genomic and epigenomic perspective, and the developmental origins of these gestational tumours are not yet well explained. Advancing our understanding of GTD could not only reduce the incidence of misdiagnosis but also provide the scientific community with deeper insights into cancer development. First, disease can exhibit a remarkable characteristic of rapid carcinogenesis, serving as a unique accelerated cancer model. Many parallels exist between tumour and early placental tissues, both of which naturally acquire immunosuppressive, invasive, angiogenic and metabolically plastic properties (reviewed by Costanzo et al., 2018). GTD could serve as a model for exploring these parallels in depth, providing insights into the mechanisms underlying shared characteristics. Second, understanding why GTD lesions exhibit high treatment responsiveness could identify factors underlying their sensitivity to therapy, potentially informing strategies for managing other, more resistant, cancers. Furthermore, current treatment modalities for severe GTN primarily involve hysterectomy or multi-agent chemotherapy with possible gonadotoxic effects, reducing the chances of successful pregnancy in the future (Joneborg et al., 2021). Additionally, a notable subset of patients (10-20%) still develop resistance to currently available therapies (Joneborg et al., 2021). Studying GTD at the molecular level has the potential to advance the development of more effective therapeutic interventions, improving outcomes for patients and informing broader cancer research efforts.

This Review provides a unique perspective on our current molecular understanding of GTD, which distinguishes it from previous work that tends to reflect on the clinical aspects of this condition. In the first section, we examine the key pathological features of GTD, focusing on its genetic origins and subtypes. In the second half of the Review, we transition to focused vignettes in which we highlight major areas that investigators have looked into to identify GTD-linked molecular signatures, including germline and *de novo* genetic changes, mitochondrial dysfunction, DNA methylation patterns and the emerging role of microRNAs (miRNAs). By integrating critical insights into genetic and epigenetic mechanisms, we aim to address the complexities of understanding the molecular drivers of GTD.

**Box 1. A short primer on the placenta**

Following zygote formation, the first cell fate decision divides embryonic cells into two lineages: the inner cell mass, which forms the embryo, and the outer trophoblast layer (the trophectoderm), which gives rise to the placenta (Piliszek et al., 2016). The placenta plays a crucial role in pregnancy, providing the conceptus with anchorage to the maternal decidua (the modified lining of the uterine endometrium that forms during pregnancy), protection from immune destruction, and the ability to exchange nutrients and waste products with the mother (Burton and Jauniaux, 2015). These essential functions are possible because of the differentiation of the trophectoderm (TE) into specialised trophoblast cell types: cytotrophoblast (CT), extravillous trophoblast (EVT) and syncytiotrophoblast (SCT) (James et al., 2012). The CT constitutes an undifferentiated proliferative lineage that can give rise to the EVT and SCT. EVT cells that invade the decidualised endometrium are called interstitial EVT, and those that transform uterine vessels are known as endovascular EVT (Cierna et al., 2016). The SCT is composed of multinucleated cells formed through the fusion of mononuclear CT cells, and it forms a thin barrier between maternal and foetal blood, thereby facilitating the transfer of nutrients and oxygen to the foetus. All three major trophoblast subpopulations arise from the TE by means of tightly controlled genetic, epigenetic and physiological regulation, as well as a complex network of interactions between the trophoblast cell populations and the surrounding decidual and immune cells (Vento-Tormo et al., 2018). Dysregulation of these processes leads to impaired trophoblast differentiation, which contributes to various complications of pregnancy, including not only GTD but also miscarriage, preterm birth, intrauterine growth restriction and pre-eclampsia (Robinson et al., 2023).

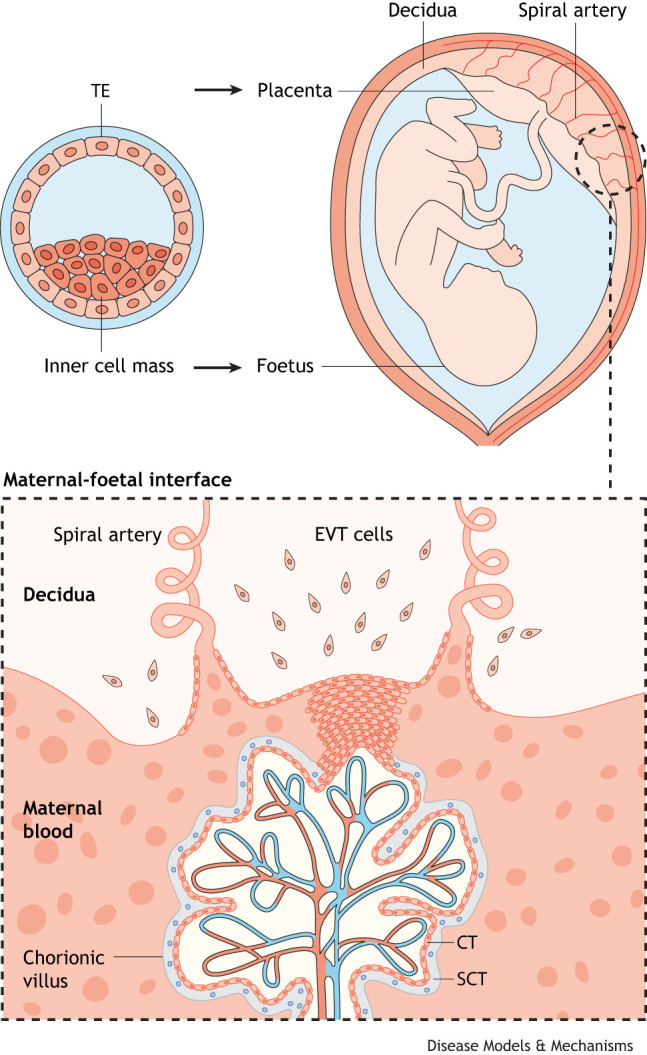

## Pathological features of GTD

Hydatidiform mole, also known as molar pregnancy, originates from the cytotrophoblast (CT; Box 1) and occurs in ∼1 in 600 pregnancies in the UK, with higher incidence in African and Asian countries (Savage et al., 2013; Eysbouts et al., 2016). Different types of trophoblastic moles have distinct genetic features (Fig. 2). PHMs are triploid; they have one set of maternal and two sets of paternal chromosomes. This type of mole typically arises because of dispermy (fertilisation of an ovum by more than one sperm; Fig. 2A), or, very rarely, fertilisation by a single diploid sperm or a haploid sperm, the genome of which is then duplicated. Sporadic CHMs, in turn, are diploid androgenetic, as they have two sets of paternal chromosomes and no maternal chromosomes. It is estimated that most CHMs originate from fertilisation of an ovum by a single haploid sperm, followed by duplication of the paternal genome (Fig. 2B, top), while approximately one quarter of CHM cases are estimated to result from dispermy (Fig. 2B, bottom) (Fisher and Maher, 2021). The mechanism and the timing of maternal chromosome loss in CHMs remain the subject of debate. One theory suggests that the ovum is anucleate at the time of fertilisation, and the likely origin of such an ‘empty egg’ is non-disjunction during meiosis, which results in loss of maternal chromosomes to one of the polar bodies (Kajii and Ohama, 1977). Recent research has provided some evidence for this hypothesis; through genetic analysis of patients with recurrent androgenetic moles, one study identified the link between CHMs and biallelic mutations in the *MEI1* gene, which plays a key role in meiotic synapsis through the formation of double-strand breaks. This work showed that, indeed, 8% of oocytes from female *Mei1^−/−^* mice were anucleate, having extruded their chromosomes together with the spindles into the first polar body (Nguyen et al., 2018). However, direct evidence for the existence of such ‘empty eggs’ in humans is still lacking. Another hypothesis argues that loss of maternal DNA occurs during post-zygotic diploidisation of a triploid conceptus (Golubovsky, 2003). Recently, support for this mechanism was provided by the demonstration that androgenetic blastomeres can arise owing to whole-genome segregation errors in both human and bovine embryos (De Coster et al., 2022).

In addition to their inherent genetic differences, CHMs and PHMs exhibit different features of embryonic development. Diploid CHMs contain no embryonic material, whereas some embryonic tissue is present in pregnancies diagnosed as PHM (Candelier, 2016). Interestingly, the female counterpart of CHMs, ovarian teratoma, has two sets of maternal chromosomes and develops tissue characteristics of the three germ layers of the embryo, but not of the extraembryonic components that give rise to the placenta (Linder et al., 1975). This observation supports the theory that paternal gene expression plays a very important role in the development of the placenta (Wang et al., 2013).

Meanwhile, APSNs, a recent addition to the GTD classification, are usually low risk and do not require treatment unless they become symptomatic. However, ∼15% of these lesions undergo malignant transformation and develop into PSTT or ETT (Kaur et al., 2015), which has justified their inclusion in the GTD classification. Thus, careful medical monitoring is needed for patients diagnosed with APSN.

On the malignant end of the disease spectrum, GTN encompasses a group of highly invasive, metastatic cancerous lesions. Although these tumours comprise a small proportion of total diagnosed GTD cases, they represent the primary cause of mortality associated with this condition (Kingdon et al., 2012; Kohorn, 2014). Invasive moles occur when a hydatidiform mole, in most cases a CHM, invades the myometrium, the muscular wall of the uterus. Approximately 1 in 14 patients who have experienced a complete molar pregnancy is estimated to subsequently develop an invasive mole (Hancock et al., 2022). CC is the most aggressive form of GTD, with an incidence of ∼1 in 40,000 pregnancies in European countries (Eysbouts et al., 2016). Interestingly, non-gestational CCs also exist, which can be of germ cell origin or associated with a somatic high-grade malignancy, and are believed to arise through retrodifferentiation, a process by which cells lose their differentiated properties and revert to a stem-like state reminiscent of early development (Uriel, 1976; Cheung et al., 2009). Like other trophoblastic tumours, PSTT is extremely rare and occurs in 1 in 100,000 pregnancies in the UK, with even lower incidence for ETT. Although all invasive moles develop from a hydatidiform mole, only ∼50% of CCs and ∼25% of PSTT and ETT cases follow a molar pregnancy, and the remainder of the cases occur following any other type of pregnancy (Hanby and Walker, 2004). Immunohistochemical analysis of protein expression patterns in gestational tumours suggest that non-molar-derived cases of GTN arise as a result of neoplastic transformation of undifferentiated CT cells early in embryonic development (Box 1). Thereafter, specific differentiation programmes are hypothesised to determine whether CC, ETT or PSTT will develop (Mao et al., 2007).

## Genetics of GTD

### Mutations in the maternal germline that increase the risk of GTD

The majority of molar pregnancies are sporadic. However, a small subset of patients has an inherited predisposition to developing hydatidiform moles, a condition known as familial recurrent hydatidiform mole. This is an autosomal recessive disorder in which most affected women develop diploid biparental hydatidiform moles (BiHMs). Although BiHMs are most often karyotypically normal (cases of digynic triploid conceptuses have been reported by Fallahian et al., 2013 and Allias et al., 2020) (Fig. 2), the molar phenotype results from imprinting defects, akin to sporadic CHMs (Williams et al., 2010). BiHMs arise owing to the presence of biallelic mutations in *NLRP7* or, less commonly, *KHDC3L* in the maternal genome. *NLRP7* encodes a protein that plays an important role in inflammation and apoptosis, and its knockdown in human embryonic stem cells has been shown to alter DNA methylation and accelerate trophoblast differentiation (Mahadevan et al., 2014). More recently, it has been shown that NLRP7 can promote HLA-C expression in trophoblasts (Gu et al., 2024). In addition, as discussed in more detail in the next section, mutations in *NLRP7* lead to loss of maternal imprinting in the trophoblast (Sanchez-Delgado et al., 2015). Meanwhile, mutations in *KHDC3L* can disrupt DNA damage repair pathways (Zhang et al., 2019) and lead to genome-wide loss of methylation in oocytes (Demond et al., 2019). Germline mutations in genes other than *NLRP7* and *KHDC3L* have been linked to recurrent molar pregnancy. For example, biallelic germline mutations in *MEI1*, *TOP6BL* and *REC114* have been linked to increased risk of recurrent androgenetic CHMs (Nguyen et al., 2018). These findings highlight the importance of clinical germline sequencing of these genes to facilitate early therapeutic intervention for susceptible patients.

### *De novo* somatic mutations in GTD

The genetic drivers of sporadic molar pregnancies and GTN remain unknown. Mutations in *TP53*, a crucial tumour suppressor mutated in a wide variety of cancer types, were observed in CHM and PHM using highly sensitive pyrosequencing, but not with Sanger sequencing (Chen et al., 1994; Cheung et al., 1994; Shi et al., 1996), indicating that these mutations occur only in a subpopulation of molar cells and are thus unlikely to be crucial in tumourigenesis (Chan et al., 2018). Targeted next-generation sequencing (NGS) of over 600 genes linked to oncogenesis identified very few somatic mutations in CC compared to non-gestational CC (Xing et al., 2019). Additional evidence for the low mutational load in the genomes of gestational CCs was provided shortly afterwards by studies that separately utilised whole-exome sequencing (WES) and whole-genome sequencing (WGS) of a single tumour (Lazare et al., 2019; Savage et al., 2019). Further, WES analysis of a larger sample size of 20 CCs identified mutations in chromatin remodelling genes (*ARID1A*, *SMARCD1* and *EP300*), although these mutations were each identified in a single sample (Jung et al., 2020). NGS analysis of one PSTT and one ETT sample identified a *TP53* mutation in PSTT, as well as missense mutations in tumour suppressors *APC* and *SMAD4* in both PSTT and ETT (Öz Atalay et al., 2023). More recently, the mutational profile of an invasive mole was analysed for the first time, along with the largest sample set of the rarest forms of GTD, comprising seven ETT and five PSTT samples, using a targeted NGS panel and WES (McNally et al., 2024). This study further supported the lack of a consistent point mutational pattern, with a third of CC samples analysed harbouring *TP53* mutations, one of seven ETT samples having an *EGFR* variant, and one of five samples exhibiting an *ERBB2* mutation, with the invasive mole lacking any clinically important alterations. Thus, the GTD mutational landscape is heterogeneous, with no clear support for a definitive mutational driver, and is likely to be driven by mechanisms other than mutations in cancer-associated genes.

### Structural alterations in GTD genomes

In addition to analysis of point mutations, large-scale genetic alterations in GTD lesions have been explored, with a particular emphasis on GTNs owing to their metastatic potential, which poses a significant risk to maternal health. Early studies reported that chromosomal rearrangements, such as 8p12-p21 deletion and 7q21-q31 amplification, occur frequently in CC, identifying these copy number alterations (CNAs) as potential markers for this type of cancer (Surti and Habibian, 1989; Ahmed et al., 2000). However, in a more recent analysis of 20 CCs, these CNAs were not found to be the predominant genetic changes (Jung et al., 2020), casting doubt on 7q21-q31 and 8p12-p21 alterations as definitive markers for gestational CC. A different study identified several CNAs (gain of 17p25 and deletions of 9q33 and 18q22) that were found in at least half of the eight analysed samples (de Mello et al., 2017). *In silico* analysis of genes affected by these CNAs pointed towards activation of the PI3K/AKT signalling pathway, which was later confirmed to be dysregulated in GTN through whole-transcriptome analysis (Cho et al., 2020). Furthermore, one of the three regions most affected in CC, gain of 17q25, mapped to the CBX family of genes (*CBX2*, *CBX4* and *CBX8*). Analysis of protein–protein interaction networks demonstrated that the proteins encoded by these genes play an important role in Polycomb repressive complexes, which are known to regulate genes through histone modification (Piunti and Shilatifard, 2021) and are crucial players in the maintenance of the placental epigenome (Box 2) (Weigert et al., 2023). In addition, the same study concluded that gestational CCs have overall higher genomic instability than non-gestational CCs (De Mello et al., 2017). Sequential analysis of a hydatidiform mole, an invasive mole, and a CC from the same patient using single-nucleotide polymorphism (SNP) assays, short tandem repeat markers and WES showed enrichment of CNAs in CC, but not in the other, less aggressive, GTD lesions, suggesting that large-scale genetic changes can be biomarkers for poor prognosis (Jung et al., 2020), a feature shared among many cancer types (Hieronymus et al., 2018). Another study, which analysed a relatively larger number of CC samples (*n*=15), identified only one specimen with a CNA, an amplification of *SS18*, a gene encoding a subunit of the chromatin remodelling complex BAF (McNally et al., 2024). Further, one ETT sample harboured amplifications of both *MYD88* and *NCKIPSD*, and another ETT had amplifications of *FGFR3* and *RALGDS*. Although limited by sample size, these studies collectively indicate the presence of intra-patient heterogeneity in GTN patients not only in the context of point mutations, but also in terms of larger-scale genetic changes (Table 1).

**Box 2. The human placenta methylome**

The placenta is a fascinating tissue from an epigenetic point of view. First, of all tissues in the human body, the placenta has the lowest level of methylation at 3.2%, compared to the highest methylation level of 4.26% observed in the brain (Tsien et al., 2002). This global hypomethylation, observed in all studied mammalian placentas, suggests a conserved functional role that has yet to be discovered (Schroeder et al., 2015). However, it remains unclear whether this hypomethylation is enriched in certain genomic regions more than others.

Another unique feature of the placental epigenome, not observed in other postnatal human tissues, is partially methylated domains (PMDs), which are large regions of reduced methylation spanning over 100 kb that are interspersed with regions of higher methylation (Schroeder et al., 2013). A recent study identified the mechanism by which the unusual epigenetic landscape of the placenta is maintained; regions of intermediate methylation are preserved by an equilibrium of two counteracting forces – continuous recruitment of *de novo* DNA methyltransferase (DNMT) enzymes and antagonising Polycomb domains that protect DNA from methylation by means of epigenetic modification of histone proteins (Weigert et al., 2023). Nevertheless, the functional role of these remarkably complex regulatory processes and the resulting placenta-specific regions of intermediate methylation remain to be elucidated.

The placental methylome is also distinct because it exhibits increased methylation of CpG islands in the promoters of developmental genes (Schroeder et al., 2013). It is now known that *de novo* methylation of these promoters is mediated by DNMT3B and is crucial for the establishment of the maternal–foetal interface (Andrews et al., 2023). However, the mechanisms promoting the acquisition of 5-methylcytosine (5mC) specifically at these sites are not yet fully understood (reviewed by Pastor and Kwon, 2022).

Although the placental methylome is strikingly different from somatic tissues, its features (global hypomethylation, PMDs, and increased methylation of promoters associated with developmental regulators) are shared with cancer (Novakovic and Saffery, 2013; Smith et al., 2017). Thus, understanding the processes that maintain the placental epigenome and how these processes become dysregulated in GTD could provide key insights into the role of DNA methylation in cancer cells.

**Table 1.** A summary of somatic genetic changes identified in GTD lesions

References	GTD samples studied	Assay	Main findings
***De novo* mutations**			
Cheung et al. (1994)	4 HMs	Sanger sequencing	None of the 4 samples had a *TP53* mutation.
Chen et al. (1994)	23 CHMs and 1 PHM	Sanger sequencing	1 of 24 samples (4.17%) had a *TP53* mutation.
Shi et al. (1996)	14 HMs, 6 invasive moles and 8 CCs	Sanger sequencing	None of the 38 samples had a *TP53* mutation.
Chan et al. (2018)	43 CHMs and 6 PHMs	Pyrosequencing	At least 1 *TP53* mutation in 36 of 49 cases (73.74%).
Xing et al. (2019)	6 CCs	NGS of 637 cancer-related genes	Mutations in the following genes occurred in 1 of 6 (16.67%) samples: *TP53*, *CDH2*, *LRP1B*, *NOTCH1* and *IDH2*.
Savage et al. (2019)	1 CC	WGS	Lack of mutations in cancer-associated genes. Deletion in *GIGYF2*, single-nucleotide variant in *LOC101929543*, *LOC101928951*, *ANKRD20A5P*, *NBPF9*, *C6*, *IQSEC3* and *IGFBP3*.
Lazare et al. (2019)	1 CC	WES	No well-known driver mutation identified, but 75 ZNF, 35 SLC and 7 MUC family genes contained a mutation.
Jung et al. (2020)	20 CCs	WES	Mutations in the following genes occurred in 1 of 20 samples (5%): *ARID1A*, *SMARCD1*, *EP300*, *AMER1*, *ZNF429*, *DNMT3A*, *NFE2L2*, *CACNA1D* and *PTCH1*.
Öz Atalay et al. (2023)	1 PSTT and 1 ETT	NGS	Mutations in *KDR*, *APC* and *SMAD4* were shared between tumours. *KIT* and *TP53* mutations were observed only in PSTT, while *PIK3CA*, *RB1* and *SMATCHB1* mutations were identified in ETT.
McNally et al. (2024)	15 CCs, 7 ETTs, 5 PSTTs, 1 invasive mole and 2 mixed histology	WES and NGS	5 (33.3%) CCs had a *TP53* mutation. 1 ETT (16.7%) had a mutation in *EGFR*, and 1 PSTT (20%) had a mutation in *ERBB2*. No driver mutations detected in the invasive mole.
**CNAs**			
Surti and Habibian (1989)	BeWo, DoSmi, ElFa and JAR cell lines	Q, G, C banding	Rearrangements of chromosomes 1, 7, 9, 10 and 12, as well as breakpoints on chromosomes 1, 3, 9, 13, 12, 7 and 21, were observed in all 4 cell lines.
Ahmed et al. (2000)	12 CCs, 5 CHMs and 2 PHMs	Comparative genomic hybridisation	HMs showed normal profiles, while 5 of 12 (41.7%) CCs had loss of 8p12-p21 and 4 of 12 (33.3%) CCs showed amplification of 7q21-q31.
Poaty et al. (2012)	10 primary CCs, and BeWo, JEG-3 and JAR cell lines	Array comparative genomic hybridisation	4 of 10 (40%) CCs exhibited 1-11 CNAs; 6 (60%) samples showed no detectable acquired CNAs.
de Mello et al. (2017)	8 CCs	Array comparative genomic hybridisation	3 cases (37.5%) had gain of 1p36.33-p36.2, 4 cases (50%) had gain of 17q25.3 (including *CBX2*, *CBX4* and *CBX8*); 5 cases (62.5%) had loss of 9q33.1 (including *TRIM32*), 3 cases (37.5%) had loss of 17q21.3, and 4 cases (50%) had loss of 18q22.1 (including *CDH19*).
Xing et al. (2019)	6 CCs	NGS of 637 cancer-related genes	Recurrent amplification of *CEBPB*, *CEBPA*, *FOXL2*, *EXOSC6*, *IRS2*, *SOCS1*, *SOX11*, *MAFB*, *MYCN*, *MYCL1*, *RICTOR*, *MNX1*, *ROS1*, *BCL11B*, *POT1*, *DST*, *LIFR* and *TOP1*.
Jung et al. (2020)	29 CCs	SNP microarray	25 of 29 samples (86.2%) had 2-22 CNAs, while 4 samples (13.8%) harboured no CNAs. The most recurrent was gain of 1q21.1-q44 (13 samples, 44.8%); gain of 12p13.33-p11.1 (including *KRAS*) was detected in 6 (20.7%) samples.
McNally et al. (2024)	15 CCs, 7 ETTs, 5 PSTTs, 1 invasive mole and 2 mixed histology	WES and NGS	Amplification of *SS18* was detected in 1 CC; 1 ETT contained amplifications of *MYD88* and *NCKIPSD*, and another ETT sample harboured amplifications of *FGFR3* and *RALGDS*.

CC, choriocarcinoma; CHM, complete hydatidiform mole; CNA, copy number alteration; ETT, epithelioid trophoblastic tumour; GTD, gestational trophoblastic disease; HM, hydatidiform mole; NGS, next-generation sequencing; PHM, partial hydatidiform mole; PSTT, placental site trophoblastic tumour; SNP, single-nucleotide polymorphism; WES, whole-exome sequencing; WGS, whole-genome sequencing.

## Mitochondrial DNA

Although CHMs lack any maternal contribution to the nuclear genome, maternal mitochondrial DNA (mtDNA) is retained. Interestingly, it has been argued that instability in mtDNA could also play a role in GTD pathogenesis. The first study to consider mitochondrial gene expression identified four downregulated transcripts in GTD compared to healthy controls, encoding COX1, ATPase subunit 6, 12S rRNA and tRNA(Phe) (Durand et al., 2001). A subsequent study, which sequenced mtDNA from ten hydatidiform moles and one CC sample, surprisingly found six somatic point mutations in the mtDNA of the tumour, in contrast to the complete absence of mutations in the hydatidiform mole samples (Man Chiu et al., 2003). This observation suggests that mutations in mitochondrial genes could play a role in the development of an invasive phenotype of GTN. Moreover, a recent study showed that increased production of reactive oxygen species in the CC-derived BeWo cell line impacts the fusion of trophoblast cells and the production of endocrine hormones by SCT, which is essential for healthy placental development (Box 1) (Walker et al., 2020). Further investigation into the importance of mitochondrial function in GTD is thus crucial, especially in relation to the progression towards the more invasive, metastatic GTN.

## Epigenetics of GTD

The role of epigenetics, and particularly DNA methylation, is clear in the context of molar pregnancy, in which a global defect in genomic imprinting is observed owing to the disrupted ratio of the maternal and paternal genomes. Genomic imprinting is a mechanism that enables the preferential expression of either the maternal or paternal allele, determined by the parent of origin and controlled by epigenetic modifications. Many epigenetic studies in the context of GTD have focused on BiHMs because they develop a molar phenotype despite having a normal biparental diploid genome. Bisulfite sequencing of familial moles with *NLRP7* mutations showed a lack of DNA methylation at several normally maternally imprinted loci, such as *PEG3*, *SNRPN*, *KCNQ1OT1*, *GNAS*, *PEG10* and *PLAGL1* (Kou et al., 2008; Hayward et al., 2009). A later study suggested that both androgenetic CHMs and biparental moles with *NLRP7* mutations showed a lack of methylation not only at maternally imprinted loci, but also at placenta-specific differentially methylated regions (Sanchez-Delgado et al., 2015). Although one would expect to observe double the expression levels of paternally expressed genes and none of the maternally expressed genes in CHMs (owing to the absence of maternal chromosomes), early GTD research contradicts this theory. Studies found that both *H19* (normally maternally expressed) and *IGF2* (normally paternally expressed) are expressed in CHMs (Mutter et al., 1993). This suggests that, in addition to deregulated allele-specific gene expression caused by imprinting abnormalities, global genome demethylation can also contribute to GTD pathogenesis.

Although some early studies focused on methylation within imprinted regions owing to their importance in placental development and function, new research has shed light on changes in methylation levels beyond these regions. A genome-wide methylation analysis of a single CC sample and matched term placenta showed that, globally, the tumour had more hypomethylated sites than did the control (Savage et al., 2019). This parallels many other cancers, which undergo non-mutational epigenetic reprogramming during malignant transformation (Hanahan, 2022). Interestingly, in one sample of CC studied, many CpG island promoters underwent hypermethylation, including some promoters of tumour suppressor genes such as *FAN1*, *ZNF471* and *ZNF671* (Savage et al., 2019). Another noteworthy observation is that the CC methylome showed the highest similarity to the first trimester placenta compared to placentas from later stages of gestation, supporting the hypothesis that GTN arises early in embryonic development (Mao et al., 2007). However, it is difficult to draw firm conclusions based on a single sample. In contrast, bioinformatic meta-analysis of methylation data from healthy placenta, molar tissue and a CC cell line showed that levels of methylation positively correlated with clinical pathology severity, with invasive CC cells exhibiting the highest levels of DNA methylation (Szabolcsi et al., 2021), suggesting that global hypermethylation could be responsible. The same study also identified the potential reason behind this trend; analysis of mRNA microarray data from a CC-derived cell line showed significantly increased expression of DNMT3B, an enzyme that is a crucial positive regulator of *de novo* methylation in the placental epigenome (Andrews et al., 2023). However, this computational analysis only included array data, which considers a much smaller number of methylation sites. Thus, there is conflicting evidence regarding the overall epigenomic status of GTD DNA, emphasising the necessity for further genome-wide analyses with larger sample sizes to elucidate the epigenetic mechanisms driving this condition.

## Emerging role of miRNAs in GTD progression

More recently, a role for miRNAs has been proposed in GTD. miRNAs are a class of small non-coding RNA molecules that play an important role in post-transcriptional gene expression. Analysis of chromosomal abnormalities in 11 CC samples and three CC-derived cell lines identified an enrichment of miRNA-encoding genes and miRNA clusters in CNAs that overlapped in at least two samples (Poaty et al., 2012). These included an miRNA cluster containing more than 50 miRNAs called C19MC, which is known to play a role in trophoblast invasion (Xie et al., 2014) and the aberrant methylation of which has been linked to hepatocellular carcinoma and non-small cell lung cancer (Rui et al., 2020; Boyero et al., 2023). This cluster was further confirmed to be aberrantly methylated in recurrent hydatidiform moles, providing additional evidence for the potential role of miRNAs in GTD pathogenesis (Sanchez-Delgado et al., 2015). The same study reported aberrant expression of the miRNA processor *LIN28B* from the maternal allele, and overexpression of this protein has been associated with poor disease outcomes in ovarian cancer (Hsu et al., 2015). Furthermore, several studies have reported abnormal miRNA expression in hydatidiform moles and CC compared to healthy placenta (Chao et al., 2010; Na et al., 2012; Hasegawa et al., 2013; Wang et al., 2017; Guo et al., 2019). However, a distinct miRNA expression signature specific to GTD has yet to be identified.

Efforts to identify miRNAs that could act as biomarkers for progression of a mole into a tumour have shown inconsistent results (Zhao et al., 2018; Lin et al., 2021; Teerapakpinyo et al., 2022). Zhao et al. (2018) suggested that differentially expressed miR-371a-5p and miR-518a-3p can induce GTN progression in moles through negative regulation of the p53 regulator BCCIP, the core transcription factor for pluripotency SOX2, and tumour suppressor MST1. Lin et al. (2021) reported elevated levels of miR-181 miRNAs in pre-GTN CHM cases compared to regressed moles. Expression of BCL2, a regulator of apoptosis, is controlled by miR-181 family members. The study further confirmed reduced BCL2 expression in pre-GTN moles at both the mRNA and protein levels, suggesting that miRNA-mediated disruption of apoptosis may contribute to GTN progression. A separate study, which focused specifically on miRNAs predominantly expressed in the trophoblast, identified decreased expression of 14q32 miRNAs and loss of DIO3 expression (confirmed by immunohistochemistry) as potential biomarkers for mole-to-cancer progression (St. Laurent et al., 2021). Given that 14q32 miRNAs, like other trophoblast-specific miRNAs, are regulated by imprinting, the authors proposed that benign moles and pre-GTN moles have different imprinting patterns, which determine whether the mole will develop into cancer.

**Fig. 3.**
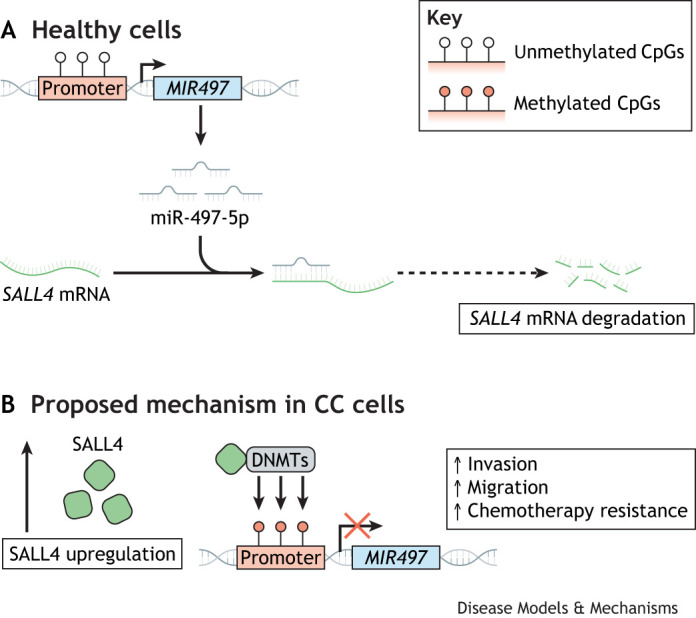
**The positive DNA methyltransferase (DNMT)–miR-497-5p–SALL4 loop, as proposed by** **Peng et al. (2021)****, allows CC cells to maintain stem-like properties.** In healthy cells (top), miR-497-5p is expressed from the *MIR497* gene and negatively regulates *SALL4* expression. In CC (bottom), it is proposed that aberrant expression of SALL4 leads to recruitment of DNMTs to the promoter of miR-497-5p, leading to its methylation, which blocks expression of the miRNA. Removal of the silencing miR-497-5p leads to increased production of the transcription factor SALL4, which allows CC cells to upregulate other pathways that are involved in invasion, migration and therapy resistance.

A recent study has identified a link between miRNA expression and epigenetic regulation in GTD. The researchers proposed a feedback loop mechanism by which CC cells acquire stemness characteristics, which are associated with increased resistance to therapy and an increased chance of relapse (Peng et al., 2021). According to the model, SALL4, a transcription factor aberrantly expressed in CC, recruits DNMT enzymes. Redundant DNMTs promote methylation of the miR-497-5p promoter, leading to miR-497-5p silencing. This, in turn, enhances *SALL4* mRNA translation by removing the suppressive miR-497-5p (Fig. 3) (Peng et al., 2021). This feedback loop, verified *in vitro* and *in vivo*, enables CC cells to maintain an ‘immortalised’ stemness. This finding further underscores the role of DNA methylation in GTD and suggests that DNMT inhibitors could represent a promising approach for therapeutic intervention in GTD.

## Conclusions and future perspectives

Despite significant advancements in our comprehension of GTD at the molecular level, the pathogenic mechanisms underlying this disease remain elusive. Given the lack of recurrent point mutations or large-scale genetic alterations observed in GTD genomes, it seems increasingly likely that GTD lesions arise through non-genetic mechanisms of cancer initiation. This phenomenon is also observed in certain paediatric cancers, for example ependymomas, which have a low mutational load and are driven by epigenetic changes that alter gene expression in ways that favour cancer progression (Mack et al., 2014). Additional evidence for the role of epigenetics in placental tumours is provided by the *SALL4* miRNA feedback loop involving DNMT enzymes. Another interesting and largely overlooked observation in GTD literature is the potential role of mtDNA mutations in GTD pathogenesis. Given the emerging link between mitochondrial metabolism and DNA methylation (F. C. Lopes, 2020), mutations in mtDNA could also contribute to the dysregulated epigenome in GTD.

It is important to highlight several open questions for further research into GTD biology. An important feature of the placental methylome that should be considered in the context of GTD is partially methylated domains, a phenomenon also observed in cancer (Box 2) (Weigert et al., 2023; Nishiyama and Nakanishi, 2021). Understanding whether these methylation patterns are preserved or disturbed in GTD lesions could shed light on global epigenetic reprogramming in this disease. Furthermore, *NLRP7*, a gene involved in genomic imprinting, has been found to be highly expressed in CHMs, CCs and a human-derived CC cell line, JEG3. In a mouse CC model, this gene has been shown to promote tumour growth through the establishment of an immunosuppressive microenvironment, which in turn downregulates the maternal immune response (Reynaud et al., 2021). Given the striking similarities between the microenvironment of trophoblast and tumours (reviewed by Krstic et al., 2022), it would be interesting to study the mechanisms by which dysregulated imprinting in GTD influences its cellular niche.

Another way in which epigenetics might drive GTD pathogenesis is through transposon activation, which has been described in lung, breast and colorectal cancers (Pradhan and Ramakrishna, 2022). One study showed selective hypermethylation of LINE-1 transposon loci in PHM (Perrin et al., 2007), whereas a later study found that LINE-1 loci become demethylated with progression to more severe CC (Lertkhachonsuk et al., 2015), a trend observed in many other cancer types (Barchitta et al., 2014). Thus, LINE-1 methylation was proposed as a predictive biomarker for mole-to-GTN progression (Lertkhachonsuk et al., 2015). However, understanding the role of transposons in GTD is limited by the lack of long-read sequencing data from these lesions. Further, unlike embryonic cells, trophoblasts do not undergo global DNA methylation at the blastocyst stage (Sanford et al., 1985). This creates a transcriptionally permissive environment that allows endogenous retroviruses (ERVs) to be highly transcribed in the human placenta (Chuong, 2013). Early research identified human ERVK transcripts in CC but not in molar pregnancy (Herbst et al., 1996). However, the role of ERVs in GTD has not been investigated in depth, highlighting it as a potential area for further study.

Research on trophoblast lesions is significantly hindered by the rarity of GTD and the challenges in obtaining GTN tissue samples owing to the high risk of haemorrhage associated with invasive collection procedures. This scarcity, combined with small cohort sizes and varying methodologies, often leads to conflicting findings, and makes it difficult to draw broad conclusions. Although genomic technologies have advanced, and more studies are applying sequencing and array-based approaches to small sample sets, the sporadic nature of these efforts has hindered the inference of reliable, robust molecular signatures from the data. To make progress in understanding this disease, future efforts may need to consider alternative sampling strategies, such as analysis of circulating tumour DNA (Openshaw et al., 2016). Additionally, the establishment of biobanks and international treatment centres presents great potential to provide access to a wider range of GTD samples (Golfier and Seckl, 2024). Through these efforts, identifying oncogenic processes driving GTD will not only enable development of novel targeted therapies against gestational neoplasms but also enrich our understanding of the role of DNA methylation in tumour initiation and development.
